# Multimodal therapeutic efficacy assessment of vagus nerve stimulation in stroke: integrated application of imaging, electrophysiological, and behavioral indicators

**DOI:** 10.3389/fneur.2026.1788506

**Published:** 2026-02-27

**Authors:** Tiancong Fu, Hui Zhang, Haoran Ma, Zhen Liu, Yujia Li, Tinghe Zhang, Ningcen Li, Jingyu Zhang, Xiyou Hu, Zelin Chen

**Affiliations:** 1 Research Center of Experimental Acupuncture Science, Tianjin University of Traditional Chinese Medicine, Tianjin, China; 2 School of Acupuncture & Moxibustion and Tuina, Tianjin University of Traditional Chinese Medicine, Tianjin, China; 3 National Clinical Research Center for Chinese Medicine Acupuncture and Moxibustion, Tianjin, China; 4 The First Affiliated Hospital of Guizhou University of Traditional Chinese Medicine, Guiyang, Guizhou, China

**Keywords:** behavioral indicators, electrophysiological, imaging, stroke, vagus nerve stimulation

## Abstract

Stroke remains a leading cause of mortality and long-term disability worldwide, and conventional rehabilitation alone frequently results in incomplete functional recovery. This review aims to establish a mechanism-informed, clinically actionable framework for quantifying the therapeutic effects of vagus nerve stimulation after stroke across complementary modalities. We synthesize evidence spanning neuroanatomical principles, mechanistic pathways, and technological development, and organize outcome measures into an integrated triad of imaging, electrophysiological, and behavioral indicators. Across studies, imaging outcomes consistently associate stimulation with reduced infarct burden, improved blood–brain barrier integrity, and enhanced circuit remodeling, whereas electrophysiological measures capture autonomic rebalancing and neural stabilization, exemplified by increased high-frequency heart rate variability and lower low−/high-frequency ratios. Behavioral outcomes indicate clinically meaningful gains, including improvements on upper-limb motor scales (with invasive stimulation frequently associated with ≥8-point increases on the Fugl–Meyer Assessment–Upper Extremity) and reductions in post-stroke spasticity (with reported 30–40% decreases in the incidence of increased tone). Safety profiles are modality dependent: implanted systems may entail procedure- and stimulation-related adverse events that are generally manageable with parameter adjustment, whereas noninvasive approaches predominantly cause transient local discomfort with no reported fatal events. Collectively, multimodal assessment provides a rigorous “structure–electrophysiology–function–behavior” evidence chain to support precise parameter optimization, standardized implementation, and scalable translation of vagus nerve stimulation for stroke rehabilitation.

## 1 Introduction

Stroke exhibits extremely high global incidence and disability rates, ranking as the world’s second leading cause of death among non-communicable diseases (1). Post-stroke functional impairments, including motor, cognitive, swallowing, and sleep disorders, severely impact patients’ quality of life and impose substantial burdens on society and families. Traditional rehabilitation training, such as physical and occupational therapy, shows limited efficacy in certain patients. However, emerging neuromodulation techniques like VNS offer novel approaches for stroke rehabilitation. VNS, initially developed for epilepsy treatment, has expanded into stroke rehabilitation, with its therapeutic efficacy demonstrating steady growth from 2004 to 2024 (2). Non-invasive approaches, particularly transcutaneous auricular VNS (taVNS), facilitate clinical implementation due to their advantages. Given the complexity of post-stroke motor, cognitive, and swallowing function recovery, multidimensional assessment is essential, as single indicators inadequately reflect VNS efficacy. Therefore, multimodal therapeutic assessment is crucial. By integrating information from imaging (structural/functional changes), electrophysiology (neural activity), and behavioral assessments (clinical applications), VNS therapeutic effects can be comprehensively analyzed from mechanism to phenotype, demonstrating significant clinical value and future potential.

In response to these needs, this review summarizes recent advances in multimodal assessment of VNS efficacy in stroke and integrates the available evidence across three domains: imaging measures characterize structural and network-level changes, including lesion repair, blood–brain barrier preservation, and circuit reorganization; electrophysiological measures capture dynamic functional states, encompassing modulation of neural activity, autonomic balance, and motor-cortical plasticity; and behavioral measures directly quantify improvements in motor, cognitive, swallowing, sleep, and affective outcomes. By integrating modalities, we propose a continuous validation pathway linking molecular mechanisms to functional recovery and an overarching “structure–electrophysiology–function–behavior” evidence framework to support precise, standardized, and long-term evaluation of post-stroke VNS. We also highlight priorities for future work, including harmonizing assessment standards, advancing intelligent implementations, and optimizing intervention timing and stimulation parameters.

## 2 Principles, mechanisms, and technological development of vagus nerve stimulation (VNS)

This section outlines the conceptual and technological foundations for multimodal evaluation of vagus nerve stimulation in stroke rehabilitation by summarizing its bidirectional neuroanatomy and contrasting implanted with transcutaneous approaches to show how target selection, fiber recruitment, and stimulation intensity govern downstream central–peripheral modulation. Within this framework, it integrates mechanistic evidence across complementary pathways, including cholinergic anti-inflammatory signaling that attenuates pro-inflammatory cytokine activity, neuromodulator release that supports network reorganization, and plasticity-related trophic cascades that drive circuit remodeling; it also summarizes molecular programs implicated in restraining apoptosis and autophagy, maintaining blood–brain barrier integrity, and promoting angiogenesis. The technological trajectory is traced from open-loop devices to closed-loop systems that link stimulation timing to physiological biofeedback (e.g., movement- or respiration-gated paradigms), enabling more precise control of inter-individual variability and dose optimization. Finally, it critically appraises translational feasibility by comparing the strengths and limitations of different modalities and synthesizing safety profiles: implanted systems may entail procedure- and stimulation-related adverse events that are typically manageable with parameter adjustment, whereas noninvasive systems primarily cause transient local discomfort with no reported fatal events. Collectively, this section provides a mechanism-informed, technology-aware rationale for precision optimization and scalable clinical deployment of vagus nerve stimulation.

### 2.1 Core principles of vagus nerve stimulation (VNS)

The vagus nerve, the tenth cranial nerve (CN X), is a mixed nerve composed of motor, sensory, and parasympathetic fibers. It has an extensive distribution, extending from the brain to the thoracic and abdominal cavities, serving as the only long-distance neural pathway connecting the brain to peripheral organs in these regions. It includes both afferent and efferent fibers. Through its bidirectional conduction function, it regulates central neurotransmitters, balances the autonomic nervous system, and modulates immune inflammation, ultimately restoring damaged functions or correcting pathological conditions (3).

Vagus nerve stimulation (VNS) is a therapeutic technique that uses an implanted device to deliver electrical signals to the vagus nerve, thereby modulating neural function. It is classified into invasive VNS (iVNS) and noninvasive VNS (tVNS) (4). Invasive VNS involves surgically wrapping electrodes around the vagus nerve trunk in the neck, with the pulse generator implanted subcutaneously in the chest. The principle involves directly stimulating the vagus nerve trunk, delivering high-intensity, stable electrical signals that can precisely regulate both central and peripheral systems. Noninvasive VNS involves placing electrodes on the auricular cavity (vagus nerve auricular branch) or on the skin of the neck, without the need for surgery. The principle is to indirectly stimulate the vagus nerve branches, delivering lower-intensity electrical signals with higher safety, primarily through the auricular branch to the central nervous system.

### 2.2 Main mechanisms of vagus nerve stimulation (VNS)

#### 2.2.1 Anti-inflammatory mechanism

VNS primarily exerts its anti-inflammatory effects through the cholinergic anti-inflammatory pathway (CAP). It releases acetylcholine (ACh) from efferent nerves, activates *α*7nAChR on immune cell surfaces, and initiates the Jak2-STAT3 signaling pathway, downregulating pro-inflammatory cytokines (TNF-α, IL-6, IL-1β) while upregulating anti-inflammatory factors. It also enhances the expression of peroxisome proliferator-activated receptor *γ* (PPAR-γ), inhibiting pro-inflammatory factors and immune cell activation. Additionally, it may regulate inflammation by affecting the hypothalamic–pituitary–adrenal (HPA) axis (5).

#### 2.2.2 Neurotransmitter regulation mechanism

VNS regulates the release of neurotransmitters such as acetylcholine (ACh), norepinephrine (NE), and serotonin (5-HT). Experimental studies by Cheng K et al. demonstrated that VNS stimulation promotes NE release, enhancing neuroregeneration and axonal plasticity in the peri-infarct region. VNS may also indirectly influence serotonergic and dopaminergic systems, thereby modulating mood and cognitive function (6).

#### 2.2.3 Mechanism of synaptic plasticity enhancement

Transcutaneous auricular VNS (taVNS) improves ischemic damage by increasing brain-derived neurotrophic factor (BDNF) expression in the hippocampus, activating TrkB receptor phosphorylation, enhancing neuronal excitability, and promoting axonal plasticity and neurogenesis. TaVNS can also activate this pathway via α7nAChR, improving long-term neural recovery. The cholinergic basal ganglia are critical for VNS-induced cortical motor plasticity. VNS effects rely on the integrity of norepinephrine, serotonin, and cholinergic neurotransmission. It must be combined with specific rehabilitation training (e.g., task-oriented training, tone-pairing training) to maximize synaptic plasticity, promoting neural circuit reorganization and functional recovery (7).

#### 2.2.4 Mechanism of inhibition of apoptosis and autophagy

VNS can regulate molecules related to apoptosis and autophagy, reducing neuronal cell death caused by ischemic damage. The anti-apoptotic effect is reflected by a reduction in Caspase-3 (pro-apoptotic protein) levels in the ischemic penumbra, through upregulation of miR-210 (via the hypoxia-inducible factor/Akt pathway) (8) and activation of lipocalin prostaglandin D2 synthase (L-PGDS), inhibiting apoptosis. Silencing miR-210 or inhibiting L-PGDS would weaken the anti-apoptotic effects of VNS. VNS can also downregulate autophagy-related proteins Beclin-1 and LC3-II, while upregulating anti-apoptotic protein Bcl-2 and downregulating pro-apoptotic protein Bax, reducing neuronal damage by inhibiting abnormal autophagic pathways (8).

#### 2.2.5 Mechanism of blood–brain barrier (BBB) protection

VNS exerts neuroprotective effects by preserving BBB integrity and reducing the infiltration of harmful substances into brain tissue. Specifically, VNS reduces blood–brain barrier permeability, protects tight junction proteins in microvessels, and decreases the expression of matrix metalloproteinase-2/9 (MMP-2/9) in activated perivascular astrocytes. It may also regulate ACh and NE levels around the BBB, alleviating neuroinflammation and indirectly maintaining BBB function.

#### 2.2.6 Mechanism of angiogenesis promotion

VNS promotes angiogenesis during the recovery phase of ischemic brain injury by regulating angiogenic factors, thereby improving blood supply to the ischemic area. Transcutaneous auricular VNS (taVNS) can increase microvascular density and endothelial cell proliferation in the peri-infarct area (3).

### 2.3 Types and characteristics of vagus nerve stimulation (VNS) technologies

Vagus nerve stimulation (VNS) technology is currently at a stage of both diversification and clinical exploration. Its types have expanded from invasive to noninvasive approaches, demonstrating potential in the treatment of various diseases, but also facing challenges in technological optimization (9) (Table 1).

**Table 1:** Comparison of VNS technologies.

Feature	Invasive VNS (iVNS)	Transcutaneous cervical VNS (tcVNS)	Transcutaneous auricular VNS (taVNS, including closed-loop CL-taVNS) (10)
Type	Invasive (surgical implantation)	Non-invasive (transcutaneous)	Non-invasive (transcutaneous, including automated closed-loop subtype)
Operation	Surgical electrode wrapping around cervical vagus nerve trunk; pulse generator implanted subcutaneously in chest	Handheld device directly contacts cervical skin; electrical signals transmit transcutaneously to cervical vagus nerve	Conventional taVNS: Electrodes placed on auricular concha stimulating ABVN; Closed-loop CL-taVNS: Automatically controlled by biofeedback signals (EMG, respiration, EEG), e.g., MAAVNS (EMG-gated, movement-paired), RAVANS (respiration-gated)
Target	Cervical vagus nerve trunk	Subcutaneous cervical vagus nerve	Auricular branch of vagus nerve (ABVN)
Mechanism	Direct electrical transmission to vagus nerve trunk, activating afferent + efferent fibers simultaneously for central-peripheral synergistic modulation	Indirect transcutaneous stimulation of cervical vagus nerve, primarily activating afferent fibers, influencing periphery through neural reflexes	Conventional taVNS: Activates auricular afferent fibers, indirectly affecting brainstem nuclei (NTS, LC), regulating autonomic nervous and inflammatory pathways CL-taVNS: Synchronizes with physiological signals (movement, respiration) for precise timing stimulation
Clinical applications	Drug-resistant epilepsy, treatment-resistant depression, refractory heart failure (FDA-approved); also used in animal models	Post-stroke motor rehabilitation, mild–moderate symptom intervention (e.g., anxiety); alternative/adjunct to iVNS in clinical practice	Conventional taVNS: Post-stroke rehabilitation, anxiety, sleep disorders; preliminary animal studies CL-taVNS: Explores linkage with movement, respiration signals; future AI integration for precision therapy
Advantages	Direct, stable stimulation; long-term continuous stimulation; programmable parameters	Non-invasive, convenient (handheld device), high safety, high patient acceptance, no surgery required	Conventional taVNS: Non-invasive, safe, simple operation, suitable for surgical non-candidates CL-taVNS: Achieves “automated convergence to ‘neural pivot’ via real-time heart rate feedback without manual intervention,” addressing traditional open-loop system side effects and healthcare dependence (11)
Disadvantages	Requires surgery with surgical risks (infection, bleeding, tracheal hematoma); numerous complications (bradycardia, neck pain, temporary vocal cord paralysis); high device costs	Weaker stimulation intensity, high individual variability, signals easily affected by skin conditions	Relatively weaker stimulation effects, high individual variability Controversial mechanisms, methodological deficiencies in clinical trials (small samples, lack of double-blind controls, inconsistent parameters, absence of VN activation biomarker assessments)

### 2.4 Safety and feasibility of vagus nerve stimulation (VNS)

#### 2.4.1 Adverse effects of implanted VNS (iVNS) and management strategies

Adverse effects are typically mild to moderate, self-limiting, and can be classified as surgery-related or stimulation-related. Common surgery-related events include vocal cord paresis, hoarseness, and surgical-site infection; less frequent events include lead fractures and transient intraoperative bradycardia. Stimulation-related adverse effects include dysgeusia, nausea, and dysphagia. Management includes voice therapy and reduced stimulation intensity for vocal cord paresis, antibiotics and wound care for infections, revision surgery for device replacement in the event of hardware failure, and adjustment of stimulation parameters (e.g., intensity and frequency) with dietary modifications for dysgeusia, nausea, or dysphagia. Transient intraoperative bradycardia is managed by temporarily suspending stimulation and administering symptomatic treatment as necessary (2).

#### 2.4.2 Adverse effects of noninvasive VNS (nVNS) and management strategies

Because nVNS is noninvasive, adverse effects are primarily local and transient. Common events include skin erythema, pruritus, and pain; less frequent events include dizziness, headache, fatigue, and asthenia. Management includes changing electrode placement, applying conductive gel, and reducing intensity to alleviate skin discomfort. For dizziness or headache, stimulation can be started at a low intensity with the patient in a seated position. For fatigue or asthenia, treatment timing can be adjusted (e.g., avoiding periods of exertion), and long-term discontinuation is usually unnecessary. No fatal adverse events have been reported. Most symptoms resolve with parameter adjustments and supportive care, and long-term irreversible injury has not been documented (12).

## 3 Application and value analysis of multimodal imaging indicators in VNS treatment for stroke

### 3.1 Four key evaluation dimensions of imaging indicators: from injury repair to clinical localization

VNS multimodal imaging assessment for stroke should center on “injury-repair-functional recovery,” classified into four dimensions: “brain injury and vascular protection,” “metabolism and neuromodulation,” “neural circuits and microscopic remodeling,” and “clinical anatomical localization,” forming a system from fundamental mechanism verification to clinical efficacy assessment.

#### 3.1.1 Brain injury and vascular protection dimension: primary validation of injury control

This dimension represents the “primary verification” of VNS efficacy, accurately quantifying cerebral infarction extent, blood–brain barrier (BBB), and associated organ damage, clarifying VNS “brain-heart protection” dual effects, primarily used for animal experimental mechanism research and clinical acute phase assessment (Table 2).

**Table 2:** Imaging modalities and outcome measures for brain Injury and vascular protection.

Imaging technique	Assessment content	References
TTC staining (2,3,5-triphenyltetrazolium chloride)	Quantifies cerebral infarction volume, directly reflecting ischemic lesion necrosis extent, verifying VNS acute neuroprotection	(13, 14)
WGA staining + toluidine blue staining	Quantifies cardiomyocyte cross-sectional area, counts inflammatory cells; assesses post-stroke myocardial pathological damage, verifies VNS cardioprotection	(13)
Dynamic contrast-enhanced MRI (DCE-MRI)	Quantifies BBB permeability, evaluates VNS vascular repair effects	(15, 16)
Immunofluorescence staining (ZO-1/occludin)	Visualizes VNS BBB ultrastructure repair, evaluates vascular protection mechanisms	(13)

#### 3.1.2 Neural function and metabolic regulation dimension: metabolic basis of functional recovery

This dimension represents “function-related verification” of VNS efficacy, monitoring brain metabolic activity and network activation, connecting “structural repair” with “functional improvement,” primarily used for clinical efficacy mechanism and treatment protocol optimization (Table 3).

**Table 3:** Imaging modalities and outcome measures for neural function and metabolic modulation.

Imaging technique	Assessment content	References
Positron emission tomography (PET, 18F-FDG)	Evaluates regional glucose metabolism, reflecting neural functional status; increased 18F-FDG uptake post-VNS directly confirms functional recovery through metabolic activation	(17)
Functional MRI (fMRI, BOLD signal)	Evaluates brain network activation and functional connectivity; VNS enhances visuomotor cortex BOLD signal, suppresses contralateral cortex overactivation; results optimize VNS protocols	(15, 18)
Nissl staining	Assesses neuronal survival numbers and arrangement; post-VNS, neurons show organized arrangement with increased survival, reflecting neuroprotective effects	(19)
Western blot detection of inflammatory pathway proteins (NF-κB, MMP-2/9)	Indicates VNS downregulates ischemic region NF-κB phosphorylation, evaluating VNS mechanisms of neural electrical activity stabilization through anti-inflammation	(23)
ELISA detection of serum IL-1β, IL-8	Post-VNS, patient serum IL-1β, IL-8 decrease, indirectly reflecting VNS anti-inflammatory effects and neural electrical activity stability correlation	(19, 20)

#### 3.1.3 Neural circuits and microscopic remodeling dimension: structural basis of functional recovery

This dimension represents “mechanism-related verification” of VNS efficacy, observing synaptic, axonal, and neural circuit connections, demonstrating microscopic mechanisms of VNS-promoted neuroplasticity, primarily used for animal experimental mechanism research (Table 4).

**Table 4:** Imaging modalities and outcome measures for neural circuit remodeling and microstructural plasticity.

Imaging technique	Assessment content	References
Transmission electron microscopy (TEM)	Visualizes synaptic repair, confirms VNS promotes synaptic maturation and neurotransmitter release efficiency, providing microstructural support for fMRI “network activation”	(20)
Laser confocal microscopy (immunofluorescence, GAP-43/NF-200)	Evaluates axonal regeneration and branching, confirms VNS promotes neural remodeling	(20)
Neuroanatomical tracing (viral tracing, e.g., PRV/FG)	Traces neural circuit connections, confirms VNS enhances motor circuit structural connectivity	(21)

#### 3.1.4 Clinical anatomical localization dimension: foundational support for clinical application

This dimension provides “fundamental assurance” for VNS clinical application, clarifying lesion location and fiber tract damage, guiding VNS surgical safety and efficacy prediction, primarily used for preoperative clinical assessment and patient selection (Table 5).

**Table 5:** Clinical imaging for anatomical localization and preoperative assessment.

Imaging technique	Assessment content	References
Computed tomography (CT/CTA)	Directly localizes hemorrhagic lesions, excludes vascular malformations, ensures subsequent VNS surgical implantation safety	(22)
Diffusion tensor imaging (DTI) fiber tractography	Evaluates nerve fiber tract damage, predicts VNS efficacy; serves as screening indicator for suitable VNS candidates	(22)

### 3.2 Advantages, disadvantages, and development prospects of imaging indicators

#### 3.2.1 Core advantages

First, in terms of comprehensive chain evaluation, VNS supports mechanistic validation and clinical evaluation through a “micro–macro” linkage, spanning molecular mechanisms, cellular function, organ function, and clinical levels. Secondly, in advancing precision treatment, VNS not only validates efficacy but also elucidates mechanisms and guides treatment optimization, allowing the transition from “standardized” to “personalized” therapy. Additionally, imaging indicators have, for the first time, confirmed the “brain protection - heart protection” linked effect of VNS, offering new directions for preventing and treating “brain-heart syndrome” after stroke.

#### 3.2.2 Major limitations

First, clinical data is scarce, with related indicators largely based on animal studies and limited human clinical trial data. Secondly, the application threshold is high due to the cost of the equipment and the need for skilled operators, which limits its implementation at the grassroots level. Furthermore, there is a lack of unified standards, with no consensus on technical parameters, making cross-study comparisons difficult. The absence of long-term follow-up data also impedes determining the duration and patterns of treatment efficacy.

#### 3.2.3 Development prospects and optimization directions

First, enhance technological integration by conducting collaborative research on multimodal imaging indicators, developing multidimensional models, and improving the precision of evaluation results. Secondly, promote standardization by developing the “VNS Stroke Imaging Assessment Guidelines,” standardizing treatment parameters, and reducing costs and technical barriers. Additionally, expand brain-heart linkage monitoring by developing new dynamic monitoring methods to address gaps in brain-heart linkage assessment.

## 4 Application and value analysis of multimodal electrophysiological indicators in VNS treatment for stroke

### 4.1 Three key evaluation dimensions of electrophysiological indicators: from neural activity to motor function

VNS multimodal electrophysiological assessment for stroke should center on “target-electrophysiology-clinical,” classified into four dimensions: “neural electrical activity modulation,” “autonomic nervous electrophysiological balance,” “motor function electrophysiological verification,” and “molecular detection indirect electrophysiological correlation,” forming a complete chain from mechanism to clinic. Unlike imaging indicators, electrophysiological indicators focus more on real-time neural signal transmission and dynamic functional status feedback, with electrical signal changes as the core nexus.

#### 4.1.1 Direct regulation of neural activity: neuroprotective validation in ischemic brain injury

This dimension addresses VNS suppression of abnormal brain electrical activity and secondary injury reduction mechanisms, directly recording cortical electrical activity, confirming VNS neuroprotective effects, primarily used for animal experimental mechanism verification and clinical critical patient assessment (Table 6).

**Table 6:** Electrophysiological measures of direct modulation of neural electrical activity.

Electrophysiological indicator	Assessment content	References
Local field potential (LFP) + Electroencephalography (EEG)	Evaluates VNS modulation of neural circuit electrical activity; VNS activates brainstem nuclei (NTS, LC) promoting norepinephrine release, providing electrophysiological foundation for enhanced neuroplasticity (e.g., synaptic remodeling)	(17, 22)
Cortical spreading depolarization (CSD/SD) monitoring	Evaluates VNS inhibition of abnormal electrical activity in ischemic penumbra, blocking infarct expansion, confirming electrophysiological stabilization	(20, 23)

#### 4.1.2 Autonomic nervous system balance dimension: validation of brain-heart axis regulation

This dimension addresses post-stroke “autonomic nervous dysfunction” (e.g., sympathetic overactivation), monitoring cardiac electrical activity and autonomic nervous tone, evaluating VNS brain-heart combined protection, providing key evidence for reducing cardiovascular complications (Table 7).

**Table 7:** Electrophysiological measures of autonomic nervous system balance.

Electrophysiological indicator	Assessment content	References
Electrocardiography (ECG)	Verifies VNS cardiac electrical activity regulation; post-VNS, heart rate fluctuation normalizes with synchronized myocardial atrophy relief, assessing safety and cardioprotection	(13)
Heart rate variability (HRV)	Quantifies autonomic nervous balance, reflecting vagal activation degree; post-VNS in stroke patients, HRV-HF increases, LF/HF decreases, correcting sympathetic overactivation, reducing hypertension and arrhythmia risks	(20)

#### 4.1.3 Electrophysiological validation of motor function: direct evidence of motor remodeling

This dimension directly relates to post-VNS motor function recovery, recording muscle electrical activity and mapping motor cortex functional topography, evaluating VNS modulation of post-stroke muscle spasticity and motor cortex plasticity, directly reflecting rehabilitation efficacy (Table 8).

**Table 8:** Electrophysiological measures for validation of motor function and cortical plasticity.

Electrophysiological indicator	Assessment content	References
Surface electromyography (sEMG)	Evaluates VNS improvement of muscle spasticity and motor coordination; VNS suppresses motor muscle group overexcitation, relieving spasticity	(16, 20)
Intracortical microstimulation (ICMS)	Maps motor cortex functional topography, evaluates VNS promotion of cortical plasticity; optimizes treatment parameters	(18)

### 4.2 Advantages, limitations, and development prospects of electrophysiological indicators

#### 4.2.1 Core advantages

Real-time Monitoring: It allows continuous recording of neural activity changes before and after VNS stimulation, offering a more intuitive reflection of therapeutic effects. Secondly, Clear Mechanistic Pathways: It clearly reveals the complete pathway of VNS action, from target activation to functional regulation. Additionally, Comprehensive Coverage: It is highly applicable to conditions such as post-stroke motor dysfunction and epilepsy, creating a closed-loop evaluation of “Target - Electrophysiology - Clinical Function.”

#### 4.2.2 Major limitations

First, Challenges in Clinical Use: Some techniques require cortical invasion, are difficult to operate, and have limited applicability. Secondly, Indirect Association Interference: Some measurements are indirect indicators, vulnerable to interference from other variables, which affects result accuracy. Furthermore, Lack of Unified Standards and Long-term Monitoring: The absence of unified evaluation standards makes cross-study comparisons difficult, and long-term dynamic monitoring data is scarce.

#### 4.2.3 Development prospects and optimization directions

First, Promote Synchronous Multi-indicator Monitoring: Conduct synchronized analysis of VNS’s synergistic effects on the “brain, muscles, and heart” to avoid the limitations of using a single indicator. Secondly, Develop Portable Devices: Create easy-to-operate portable devices, reduce costs, and promote their adoption in community hospitals. Additionally, Establish Unified Evaluation Standards: Develop the “VNS Stroke Electrophysiological Evaluation Guidelines,” standardizing parameter settings and result interpretation criteria.

## 5 Application and value analysis of multimodal behavioral indicators in VNS treatment for stroke

### 5.1 Multidimensional functional evaluation of behavioral indicators: targeting core post-stroke impairments

VNS behavioral indicators for stroke should encompass motor function, cognitive function, swallowing function, sleep function, activities of daily living, and quality of life assessments. Unlike electrophysiological indicators focusing on real-time neural signal transmission and dynamic functional status, behavioral indicators directly center on patients’ actual performance and living conditions, comprehensively reflecting treatment impacts on daily life. Compared to imaging indicators presenting structural-functional characteristics through “micro–macro” linkage, behavioral indicators emphasize clinical practicality with high universality and simple operation, reflecting actual effects on core disability improvement and quality of life enhancement, though lacking mechanistic depth of imaging and electrophysiological indicators (Table 9).

1. Motor function assessment: measuring limb function remodeling
2. Cognitive function assessment: indicating neurocognitive protective effects
3. Swallowing function assessment: enhancing post-stroke dysphagia
4. Sleep function assessment: modulating sleep-related brain activity
5. Emotional function assessment: reducing post-stroke depression and anxiety
6. Overall function assessment: comprehensive evaluation of treatment benefits

**Table 9:** Behavioral outcome measures for motor function.

Behavioral indicator	Assessment content	References
Fugl-Meyer Assessment (FMA) (including FMA-UE upper limb, FMA-LE lower limb)	“Gold standard” quantitative indicator for VNS motor efficacy, directly correlating with neural circuit remodeling, providing core evidence for clinical decisions	(15, 24)
Wolf Motor Function Test (WMFT)	Evaluates upper limb functional activities (grasping, object manipulation); post-VNS score elevation confirms motor function improvement translates to actual activity capacity	(20, 25)
Functional Test for the Hemiplegic Upper Extremity (FTHUE)	Grades upper limb functional activity capacity (I-V grades, V optimal); post-VNS grade elevation confirms hemorrhagic stroke motor function repair	(22)
Muscle strength and tone grading	Assesses muscle strength (0-V grades) and tone (normal/increased/decreased); increased tone common in post-stroke spasticity; post-VNS, affected upper limb strength improves 1–2 grades, increased tone incidence decreases 30–40%	(22)
Forelimb grasping test (animal experiment)	Evaluates upper limb motor strength and coordination; post-VNS, rat forelimb grasping success rate significantly increases, confirming motor circuit remodeling promotion	(20)
Balance beam test (animal experiment)	Evaluates balance and gait stability, reflecting lower limb function; post-VNS, rats show shortened beam crossing time with reduced errors	(20)
Barthel Index (BI)	Evaluates activities of daily living independence (feeding, dressing); post-VNS, BI scores significantly improve with notable feeding improvement, directly enhancing self-care ability	(21)
Functional Independence Measure (FIM)	Evaluates refined daily living and social functions; post-VNS, FIM scores increase, with “social communication” improvement suggesting patient transition to social participation	(18)
Modified Rankin Scale (mRS)	Evaluates overall functional recovery and disability degree (0–6 grades, 0 normal)	(15)

### 5.2 Advantages, limitations, and development prospects of behavioral indicators

#### 5.2.1 Core advantages

First, Practical and Intuitive Efficacy: Focused on clinical symptom improvements, directly reflecting the therapeutic effect on quality of life. Secondly, High Universality: Simple to operate and suitable for use in primary healthcare settings without requiring expensive equipment. Additionally, Comprehensive Coverage: It covers both physiological functions, such as motor and cognitive, and psychological functions, such as emotions and sleep, comprehensively addressing core post-stroke impairments (Table 10).

**Table 10:** Behavioral outcome measures for cognitive function.

Behavioral indicator	Assessment content	References
Montreal Cognitive Assessment (MoCA)	Evaluates cognitive function (memory, attention); post-VNS combined with cognitive training, MoCA scores improve, confirming overall cognitive function enhancement	(15, 26, 27)
Morris water maze (animal experiment)	Evaluates cognitive function (memory, attention); post-VNS, rats show shortened escape latency with 50% increased platform crossings, confirming cognitive protection	(20)
Fear conditioning test (animal experiment)	Records fear response intensity and duration, comprehensively reflecting VNS cognitive function modulation	(20)

#### 5.2.2 Major limitations

First, Subjective Bias: Some indicators depend on patients’ subjective assessments, which can lead to biased results. Secondly, Lack of Long-term Monitoring: It is challenging to capture the long-term patterns of treatment efficacy. Furthermore, Lack of Unified Standards: The existence of multiple scales for the same functional dimensions complicates cross-study comparisons. Additionally, Weak Mechanistic Association: It cannot directly reveal the treatment mechanism, relying on imaging and electrophysiological indicators to provide additional evidence (Table 11).

**Table 11:** Behavioral outcome measures for swallowing function.

Behavioral indicator	Assessment content	References
Water swallowing test	Evaluates post-stroke dysphagia, preventing aspiration; post-VNS, swallowing scores improve, confirming swallowing function enhancement	(20, 28)
Swallowing function scale	Quantitative assessment comprehensively reflecting VNS swallowing neuromuscular function repair beyond symptom relief	(20, 29)

#### 5.2.3 Development prospects and optimization directions

First, Promote Standardization: Develop the “VNS Stroke Behavioral Indicator Assessment Guidelines” to establish unified evaluation pathways and criteria. Secondly, Develop Home Monitoring Tools: Create mobile apps and other dynamic monitoring tools for home use, enabling patients to operate them independently and facilitating long-term monitoring. Additionally, Strengthen Multi-modal Integration: Combine with imaging and electrophysiological indicators to improve the accuracy and reliability of results (Table 12).

**Table 12:** Behavioral outcome measures for sleep and mood.

Behavioral indicator	Assessment content	References
Pittsburgh Sleep Quality Index (PSQI)	Evaluates sleep quality; post-VNS, PSQI scores significantly decrease, indicating sleep regulation brain region functional improvement	(12, 15, 30)

## 6 Differences in clinical usability and operational convenience across evaluation modalities and translational solutions

The three evaluation modalities differ significantly in clinical adaptability, operational thresholds, and dissemination potential. These differences directly affect the efficiency of translating multimodal assessment into clinical practice. Identifying and addressing these key differences is essential for advancing the precision rehabilitation of VNS in stroke treatment (Table 13).

**Table 13:** Behavioral outcome measures for global function.

Behavioral indicator	Assessment content	References
Hamilton Depression Rating Scale (HAMD) (including HAMD-17)	Evaluates post-stroke depression (PSD), anxiety; 4 weeks post-VNS PSD treatment, HAMD-17 decreases, HADS scores decrease with synchronized BDNF upregulation, confirming neuroprotection improves mood, enhancing comprehensive efficacy	(12, 20)
Hamilton Anxiety Rating Scale (HAMA)		(17, 18)
Hospital Anxiety and Depression Scale (HADS)		(15)

### 6.1 Core comparison of differences (based on clinical practice scenarios)

#### 6.1.1 Behavioral assessment

The “Primary Modality” in Clinical Practice: In terms of clinical usability, it is highly versatile, applicable in settings from tertiary hospitals to primary healthcare institutions. It can be used for acute-phase screening, chronic-phase follow-up, and home monitoring without the need for complex equipment. In terms of operational convenience, it is simple to use, requiring only the evaluator to master standardized scales (e.g., FMA score, Wakita water swallowing test). Some scales (e.g., PSQI, MoCA) can be completed by the patient independently or with family assistance, and the assessment takes only 5–15 min per scale. Additionally, key limitations include: subjective bias, as some scales (e.g., HAMD) depend on evaluator experience; lack of real-time monitoring, hindering the capture of functional fluctuations during treatment; and the existence of multiple scale versions for the same functional dimension, complicating cross-study comparisons (Table 14).

**Table 14:** Key differences in clinical use across the three assessment modalities.

Behavioral indicator	Assessment content	References
National Institutes of Health Stroke Scale (NIHSS)	Evaluates VNS overall stroke damage improvement, distinguishing acute and chronic efficacy	(17)
36-Item Short Form Health Survey (SF-36)	Multidimensional scoring reflecting VNS progression from functional disability recovery to social participation improvement	(17)
ICF framework “impairment-activity-participation” three-level indicators	Comprehensively evaluates “physiological function-daily activities-social participation”; supports long-term follow-up, verifying efficacy persistence	(31)

#### 6.1.2 Imaging assessment

The “Precision Modality” Driven by Research: In terms of clinical usability, it is moderate, mainly available in tertiary hospitals or research institutions. Primary healthcare facilities cannot afford the high costs of equipment (e.g., MRI machines costing millions) and maintenance, limiting their ability to provide these resources. It is only suitable for key evaluations (e.g., preoperative lesion localization, 3–6 month postoperative structural remodeling validation) and cannot be used frequently. In terms of operational convenience, it is low; specialized technicians (e.g., radiologists, technicians) are required to operate the equipment and interpret results. Patients must cooperate during the scan (e.g., fMRI requires stillness), and some critically ill or conscious-impaired patients may not tolerate the procedure. The examination is time-consuming (15–30 min per scan) and involves a complex process. Core limitations include: high equipment and technical thresholds, hindering widespread use; lack of unified evaluation parameters (e.g., fMRI BOLD signal analysis thresholds); and radiation exposure (e.g., CT), which limits repeated use in the acute phase.

#### 6.1.3 Electrophysiological assessment

The “Supplementary Modality” with Limited Application Scenarios: In terms of clinical usability, it is relatively low. Invasive techniques (e.g., ICMS, cortical electrode implantation) are restricted to research or intensive care settings. Non-invasive techniques (e.g., EEG, sEMG) are clinically applicable but require additional equipment (e.g., multi-channel electromyography devices), which are available in fewer than 30% of primary healthcare facilities. In terms of operational convenience, it is moderate. Non-invasive techniques are relatively simple to operate (e.g., sEMG electrode attachment), but data interpretation (e.g., HRV LF/HF ratio analysis) requires expert analysis. Some techniques (e.g., CSD monitoring) demand high patient cooperation, and movement or agitation may affect signal quality. Additionally, core limitations include: high risks associated with invasive techniques (e.g., infection, nerve damage), limiting clinical applications; non-invasive techniques are vulnerable to environmental interference (e.g., EEG affected by electromyographic artifacts); and the lack of standardized data collection and analysis processes, causing significant variability in results across institutions.

### 6.2 Key implementation strategies

First, strengthen training for primary healthcare personnel, focusing on core scale usage, simple electrophysiological equipment operation, and data interpretation. This can be accomplished through online courses and hands-on practice, lowering the learning threshold. Secondly, promote healthcare insurance policy support by incorporating essential evaluation equipment (e.g., portable sEMG devices, digital scale tools) for primary healthcare into the insurance procurement list, thus reducing healthcare facility configuration costs. Additionally, establish a multi-center data-sharing platform to standardize evaluation data formats for the three modalities, and optimize result interpretation using AI algorithms (e.g., automatic recognition of fMRI neural circuit activation patterns), reducing dependence on specialists.

Through these differentiated optimization and integration strategies, multimodal assessment can transition from “research-specific scenarios” to “routine clinical applications,” preserving the precision advantages of imaging and electrophysiology while leveraging the widespread applicability of behavioral assessments, thereby truly serving the individualized rehabilitation needs of VNS for stroke treatment.

## 7 Integrated application and advantages of multimodal assessment

Multimodal assessment integration represents the core support for VNS stroke treatment progression from “empirical therapy” to “precision therapy,” integrating imaging, electrophysiology, and behavioral indicators to form a complete “microstructure-neural activity-clinical functional manifestation” assessment system rather than independent single-directional judgment. Its core value lies in overcoming single indicator limitations, achieving “1 + 1 + 1 > 3” synergistic effects, completely and accurately reflecting VNS stroke treatment efficacy, verifying mechanisms, and guiding clinical individualized therapy. Multimodal assessment integration spans the “mechanism research → clinical translation → postoperative rehabilitation” complete chain, with different indicator combinations for various scenarios but consistently targeting precise efficacy verification, fundamental mechanism clarification, and clinical individualized guidance.

Imaging indicators intuitively present structural changes, physiological indicators reveal molecular mechanisms, and behavioral indicators reflect clinical functional changes. Different modalities complement each other, retaining respective advantages while synergistically addressing problems. In Wang Y et al.’s experiment, imaging (TTC staining) indicated VNS reduced cerebral infarction volume, physiology (ELISA) confirmed VNS decreased brain IL-1β and chymase expression, and behavior (open field test) demonstrated motor ability enhancement, collectively confirming VNS achieves neuroprotection and motor function improvement through mast cell degranulation inhibition and neuroinflammation reduction (19), providing comprehensive mechanism analysis from microscopic molecules to macroscopic functions. Imaging and electrophysiological indicators stratify and exclude anatomical variable interference, enhancing behavioral indicator reliability (32).

Post-stroke involves diverse disabilities across physiological and psychological functions at different disease stages; single indicators inadequately provide comprehensive coverage. Integrated multimodal assessment evaluates VNS effectiveness not only through imaging-indicated structural repair but also electrophysiology-indicated electrical activity stabilization and behavior-indicated independent living capacity translation, covering complete disease progression and meeting different stroke stage treatment efficacy needs. In Cai X et al.’s experiment, acute stroke phase imaging (TTC staining, DCE-MRI) assessed cerebral infarction and BBB damage, physiological indicators (SD monitoring, inflammatory factors) monitored disease progression; chronic phase behavioral indicators (FMA-UE, mRS) evaluated long-term functional recovery, imaging (viral tracing, immunofluorescence) verified neural functional remodeling, satisfying different treatment stage assessment requirements (23).

In the acute phase (within 1–2 weeks of onset), VNS has been investigated primarily for neuroprotection, aiming to reduce infarct size and mitigate secondary injury. However, human evidence remains limited, and key challenges include defining the optimal treatment window, optimizing stimulation parameters, and elucidating interactions with standard-of-care therapies. In the chronic phase (≥3 months after onset), research has focused on functional rehabilitation, with VNS proposed to improve motor and cognitive outcomes by promoting neural circuit remodeling. This focus currently predominates in the clinical literature. This imbalance has hindered the translation of VNS across the full stroke care continuum. Targeted acute-phase studies are needed to address the current paucity of human data (22, 23, 33).

Simultaneous “safety-efficacy” assessment occurs. In Cheng K’s experiment, physiological indicators (heart rate assessment) confirmed VNS presents no severe cardiovascular risks, imaging indicators (MRI) detected no new brain damage, behavioral indicators (FMA-UE, MAL) showed sustained functional enhancement, strengthening VNS progression from basic research to clinical application (6). In clinical individualized guidance, multimodal assessment achieves accurate preoperative patient screening, intraoperative safety monitoring, and postoperative recovery follow-up, avoiding single indicator errors. Preoperative screening excludes ineffective patients predicting efficacy; intraoperative monitoring prevents other nerve damage enabling timely parameter adjustment; postoperative assessment evaluates functional recovery and long-term effectiveness. Wang Y et al.’s experiment demonstrated that post-treatment and 4-week follow-up imaging (SVF) showed sustained swallowing residue and aspiration improvement, with behavioral scales (MASA, FCM, RAS) maintaining superior levels versus sham stimulation, consistently confirming VNS long-term efficacy through dual-modality indicators (34). Francisco GE et al. initiated three-year efficacy follow-up evaluation, with basic motor impairment (FMA-UE) continuously improving, actual activity capacity (WMFT) synchronously enhancing, and patient quality of life (SIS-Hand) significantly increasing, confirming VNS not only repairs motor function but enhances quality of life beyond short-term compensatory effects (33).

Different stroke patients exhibit varying lesion locations and stroke types; integrated multimodal assessment enables precise treatment protocol development for individualized therapy. In Abdullahi A et al.’s experiment, multimodal indicator analysis revealed stimulation parameter (frequency, intensity) and stroke stage (acute, chronic) influences on efficacy. For instance, chronic stroke patients showed more pronounced invasive VNS efficacy, while acute patients demonstrated superior non-invasive VNS efficacy and safety, providing evidence for individualized protocol selection. Different disability types require different indicator selections (35). In Li L et al.’s experiment, upper limb motor disability selected behavioral indicators (FMA-UE, WMFT) verifying functional improvement, imaging indicators (fMRI) observing motor cortex activation, and physiological indicators (BDNF, VEGF) analyzing neurovascular repair mechanisms. Post-stroke sleep disorders selected behavioral indicators (PSQI) evaluating sleep improvement, imaging indicators (BOLD-fMRI) showing enhanced connectivity, and physiological indicators (heart rate variability) confirming autonomic nervous balance; cognitive disorders selected behavioral indicators (UFM sensory subitems) evaluating sensory recovery, imaging indicators (fMRI) observing hippocampal activation, and physiological indicators (norepinephrine) explaining cognitive modulation mechanisms (36).

Promoting VNS translation from animal experiments to human clinical application provides strong assurance. In Cai X et al.’s animal experiments, imaging (TEM, viral tracing) and physiology (SD monitoring) provided mechanistic research foundations; human clinical studies employed behavior (FMA-UE, mRS) and imaging (DCE-MRI) verifying efficacy, enhancing VNS clinical application credibility (23).

## 8 Conclusion and prospects

As a multi-target, multi-effect neuromodulation technique, VNS demonstrates significant potential in stroke functional rehabilitation, with current clinical applications covering motor, cognitive, swallowing, and other functional disabilities. Imaging, electrophysiological, and behavioral indicators reflect VNS stroke treatment efficacy from different perspectives: imaging reveals brain structural and functional remodeling, electrophysiology manifests neural activity changes, and behavioral assessment evaluates clinical functional improvement. Their integrated application deeply analyzes VNS action mechanisms, provides evidence for individualized treatment protocol development, offers strong support for VNS clinical promotion, and suits different ages and lesions.

Invasive vagus nerve stimulation (iVNS) and transcutaneous vagus nerve stimulation (tVNS), including taVNS and tcVNS, differ in their mechanisms of action, efficacy profiles, and clinical indications;distinguishing between them is crucial for informed clinical decision-making. iVNS delivers stimulation to the vagal trunk through surgically implanted electrodes, activating both afferent and efferent fibers, providing higher-intensity and more stable stimulation. As a result, it may exert stronger anti-inflammatory and neuroprotective effects, making it better suited for patients in the chronic phase (≥3 months after onset) and those with severe impairments. In multimodal assessments, iVNS is frequently associated with more pronounced structural remodeling on neuroimaging, more consistent improvements in electrophysiological indices (e.g., LFP and HRV), and greater gains on behavioral scales (e.g., ≥8 points on the FMA-UE) (1). In contrast, tVNS stimulates auricular or cervical vagal branches via cutaneous electrodes and is noninvasive, convenient, and generally safe, with high patient acceptability. It may be more appropriate for the acute phase (within 1–2 weeks after onset), patients with mild-to-moderate impairment, and use in primary care settings. Since tVNS targets only a subset of vagal fibers, it may require higher stimulation intensity and longer treatment to achieve comparable efficacy. Multimodal assessments typically reveal gradual improvements in behavioral outcomes, modest modulation of electrophysiological signals, and slower emergence of vascular protection and neural remodeling on imaging. These approaches are not interchangeable; rather, they are complementary options chosen based on disease stage, impairment severity, and healthcare resource availability (15, 33).

To date, all positive findings have been reported for VNS combined with rehabilitation (e.g., task-oriented training, robot-assisted rehabilitation, or conventional physiotherapy), indicating that rehabilitation is a critical prerequisite for VNS efficacy. In contrast, implanted VNS (iVNS) delivers electrical stimulation to the vagal trunk through surgically implanted electrodes, activating both afferent and efferent fibers. Compared with noninvasive approaches, iVNS provides higher-intensity, more stable stimulation and may yield stronger anti-inflammatory and neuroprotective effects, making it better suited for the chronic phase (≥3 months after onset) (22, 29, 34).

Although VNS shows promise in stroke rehabilitation, it should not be regarded as an effective stand-alone therapy. To date, positive findings have primarily been reported for VNS combined with rehabilitation (e.g., task-oriented training, robot-assisted rehabilitation, or conventional physiotherapy), indicating that concomitant rehabilitation is a critical prerequisite for clinical benefit. VNS administered without rehabilitation may produce transient neuromodulatory effects but is unlikely to induce durable neural circuit remodeling or sustained functional recovery.

Currently, imaging and physiological indicators predominantly involve animal experiments with limited human research data, necessitating strengthened translation to human studies while addressing standardization, correlation, parameter optimization, and intervention timing issues, expanding sample sizes and research dimensions, enhancing precision, and promoting standardized clinical multimodal assessment application. Comparative research between invasive and non-invasive VNS should fill direct comparison data gaps, clarifying optimal protocols for different strokes through multimodal indicators and strengthening long-term follow-up evaluating safety through physiological and imaging indicators, developing adverse event prevention strategies. Li JN’s article mentioned superior immediate post-taVNS rehabilitation efficacy without clarifying optimal intervention timing; future research should strengthen studies combining behavioral-physiological-imaging indicators determining accurate acute stroke VNS treatment windows (37).

Large-scale, multicenter randomized controlled trials are needed to confirm the robustness of vagus nerve stimulation efficacy across diverse stroke populations and functional domains. These studies should also identify the subgroups most likely to benefit, thereby generating high-level evidence to inform stratified clinical care (2, 6, 8).

Current evidence is limited, and several key controversies persist. Limited validation of mechanistic hypotheses: many proposed mechanisms rely primarily on animal studies and lack direct confirmation in large-scale human trials, leaving their translational relevance unclear. Methodological limitations: most randomized controlled trials (RCTs) are small, and some provide insufficient details on randomization and face challenges in blinding. Unresolved questions: consensus is lacking on whether VNS exhibits a “ceiling effect” (i.e., diminishing incremental benefit after prolonged treatment), whether responses differ between stroke subtypes (ischemic vs. hemorrhagic) due to underlying pathophysiology, and how stimulation parameters interact with age and comorbidities (6). Moreover, the lack of validated biomarkers to predict response remains a significant barrier to the clinical translation of VNS (8). Candidate electrophysiological markers include heart rate variability (HRV) as an indicator of vagal activation, vagus-nerve evoked potentials (VEP) to assess pathway integrity, and band-specific local field potential (LFP) power as a correlate of neural circuit plasticity (23). Candidate imaging markers include diffusion tensor imaging (DTI) metrics such as fractional anisotropy (FA), which may predict white-matter tract recovery potential, and fMRI BOLD activation patterns that reflect regional functional responses. These markers could support three applications: (i) pre-treatment stratification to identify patients most likely to benefit; (ii) parameter optimization during therapy by adjusting stimulation frequency and intensity using real-time VEP and HRV feedback to balance efficacy and adverse effects; and (iii) post-treatment monitoring to assess response durability early by tracking changes in imaging-derived and electrophysiological measures, thereby minimizing ineffective treatment. Advancing this line of research could enhance the precision of VNS therapy and accelerate the transition from standardized interventions to personalized treatments (19, 33).

In addition, based on the above content, we have identified the relevant key unresolved issues (Research Priorities).

1. Optimizing stimulation parameters: standardized settings for VNS frequency, intensity, pulse width, session duration, and treatment schedules are currently unavailable. Optimal parameter sets should be defined based on stroke type (ischemic vs. hemorrhagic), disease stage (acute vs. chronic), and impairment domain (motor, cognitive, and swallowing), considering interactions with age, lesion location, and comorbidities (7).
2. Identifying biomarkers for VNS response: biomarkers that predict treatment benefits and monitor therapeutic response are still lacking. Future studies should evaluate blood-based markers (e.g., inflammatory and neurotrophic factors), cerebrospinal fluid markers (e.g., neurotransmitter metabolites), and imaging-derived features (e.g., DTI indices of tract integrity) to facilitate pre-treatment stratification and real-time monitoring during therapy.
3. Comparing VNS modalities and defining target populations: comparative evidence on the efficacy and safety of implanted VNS (iVNS) versus noninvasive approaches (taVNS/tcVNS) remains limited. Key questions include whether chronic stroke patients benefit more from iVNS, whether taVNS is preferable in the acute phase, and in which scenarios closed-loop CL-taVNS outperforms conventional taVNS (23).
4. Standardizing integrative analyses for multimodal assessment: multimodal datasets are still often reported descriptively rather than analyzed quantitatively. Quantitative integration frameworks (e.g., structural equation modeling or AI-based fusion) are required to estimate the relative contributions of imaging, electrophysiological, and behavioral measures, and to establish a scalable “mechanism–structure–function” assessment workflow (5, 35).

With societal development, future research may develop portable wearable devices and smartphone apps integrating multimodal data, synchronously monitoring physiological indicators and behavioral data, combining remote imaging equipment for real-time multimodal VNS treatment monitoring with timely parameter adjustment, enhancing treatment precision. Multimodal assessment will become more convenient and intelligent; based on existing multimodal data, AI prediction models can be constructed, further promoting VNS popularization and optimization in stroke treatment. Existing studies evaluating multimodal assessment of vagus nerve stimulation (VNS) in stroke provide a foundation for clinical translation (6, 31). However, limitations in study design, sample size, and evidence quality warrant cautious interpretation of these findings in terms of reliability and generalizability.

## References

[1] Feigin VL Brainin M Norrving B Martins SO Pandian J Lindsay P . World stroke organization: global stroke fact sheet 2025. Int J Stroke. (2025) 20:132–44. doi: 10.1177/17474930241308142, 39635884 PMC11786524

[2] Deng J Yang Z Wang QM Lv ZG. Two decades of vagus nerve stimulation for stroke: a bibliometric analysis. Front Neurol. (2025) 16:1531127. doi: 10.3389/fneur.2025.1531127, 40255891 PMC12006007

[3] Austelle CW Cox SS Wills KE Badran BW. Vagus nerve stimulation (VNS): recent advances and future directions. Clin Auton Res. (2024) 34:529–47. doi: 10.1007/s10286-024-01065-w, 39363044 PMC11543756

[4] Baig SS Dorney S Aziz M Bell SM Ali AN Su L . Optimizing non-invasive vagus nerve stimulation for treatment in stroke. Neural Regen Res. (2025) 20:3388–99. doi: 10.4103/NRR.NRR-D-24-00945, 39665799 PMC11974653

[5] Liu YL Wang SR Ma JX Yu LH Jia GW. Vagus nerve stimulation is a potential treatment for ischemic stroke. Neural Regen Res. (2023) 18:825–31. doi: 10.4103/1673-5374.350698, 36204850 PMC9700118

[6] Cheng K Wang Z Bai J Xiong J Chen J Ni J. Research advances in the application of vagus nerve electrical stimulation in ischemic stroke. Front Neurosci. (2022) 16:1043446. doi: 10.3389/fnins.2022.1043446, 36389255 PMC9650138

[7] Andalib S Divani AA Ayata C Baig S Arsava EM Topcuoglu MA . Vagus nerve stimulation in ischemic stroke. Curr Neurol Neurosci Rep. (2023) 23:947–62. doi: 10.1007/s11910-023-01323-w, 38008851 PMC10841711

[8] Qi R Wang M Zhong Q Wang L Yang X Huang B . Chronic vagus nerve stimulation (VNS) altered IL-6, IL-1β, CXCL-1 and IL-13 levels in the hippocampus of rats with LiCl-pilocarpine-induced epilepsy. Brain Res. (2022) 1780:147800. doi: 10.1016/j.brainres.2022.147800, 35074405

[9] Chen Z Liu K. Mechanism and applications of Vagus nerve stimulation. Curr Issues Mol Biol. (2025) 47:122. doi: 10.3390/cimb47020122, 39996843 PMC11854789

[10] Wang W Li R Li C Liang Q Gao X. Advances in VNS efficiency and mechanisms of action on cognitive functions. Front Physiol. (2024) 15:1452490. doi: 10.3389/fphys.2024.1452490, 39444752 PMC11496278

[11] Mathews RP Habibagahi I Jafari Sharemi H Challita R Cha S Babakhani A. A closed loop fully automated wireless vagus nerve stimulation system. Sci Rep. (2025) 15:27856. doi: 10.1038/s41598-025-11159-8, 40739156 PMC12311178

[12] Chen D Zhu L Liu L Zhou D Wu X. Dynamic changes in comorbid conditions following vagus nerve stimulation for epilepsy. Acta Epileptol. (2025) 7:33. doi: 10.1186/s42494-025-00222-6, 40448234 PMC12123979

[13] Tan Q Ruan Y Wu S Jiang Y Fu R Gu X . Vagus nerve stimulation (VNS) inhibits cardiac mast cells activation and improves myocardial atrophy after ischemic stroke. Int Immunopharmacol. (2024) 139:112714. doi: 10.1016/j.intimp.2024.112714, 39068751

[14] Chang JL Coggins AN Saul M Paget-Blanc A Straka M Wright J . Transcutaneous auricular Vagus nerve stimulation (tAVNS) delivered during upper limb interactive robotic training demonstrates novel antagonist control for reaching movements following stroke. Front Neurosci. (2021) 15:767302. doi: 10.3389/fnins.2021.767302, 34899170 PMC8655845

[15] Liu M Liu M Zhang B Fang M Chen K Zhang Y . Research hotspots and frontiers of vagus nerve stimulation in stroke: a bibliometric analysis. Front Neurosci. (2024) 18:1510658. doi: 10.3389/fnins.2024.1510658, 39723424 PMC11668697

[16] Zhang J Zhou F Roberts N Yuan Q Wang M Wu G . Invasive or non-invasive vagus nerve stimulation modulation of brain function and remodeling after stroke: a review. Int J Surg. (2025) 111:2148–61. doi: 10.1097/JS9.0000000000002173, 39715153

[17] Xu J Liu B Shang G Liu S Feng Z Zhang Y . Deep brain stimulation versus vagus nerve stimulation for the motor function of poststroke hemiplegia: study protocol for a multicentre randomised controlled trial. BMJ Open. (2024) 14:e086098. doi: 10.1136/bmjopen-2024-086098, 39384245 PMC11474896

[18] Chen L Gao H Wang Z Gu B Zhou W Pang M . Vagus nerve electrical stimulation in the recovery of upper limb motor functional impairment after ischemic stroke. Cogn Neurodyn. (2024) 18:3107–24. doi: 10.1007/s11571-024-10143-8, 39555282 PMC11564590

[19] Wang Y Tan Q Pan M Yu J Wu S Tu W . Minimally invasive vagus nerve stimulation modulates mast cell degranulation via the microbiota-gut-brain axis to ameliorate blood-brain barrier and intestinal barrier damage following ischemic stroke. Int Immunopharmacol. (2024) 132:112030. doi: 10.1016/j.intimp.2024.112030, 38603861

[20] Hu Y Xiong R Pan S Huang K. A narrative review of vagus nerve stimulation in stroke. J Cent Nerv Syst Dis. (2024) 16:11795735241303069. doi: 10.1177/11795735241303069, 39677973 PMC11645777

[21] Vannemreddy PS Cummings M Bahrii RV Slavin KV. Vagus nerve stimulation in stroke management: brief review of evolution and present applications paired with rehabilitation. Brain Sci. (2025) 15:346. doi: 10.3390/brainsci15040346, 40309799 PMC12025364

[22] Xia C Xu P Wang L Zhang D Qi Y Wu M . Vagus nerve stimulation combined with nerve rehabilitation therapy for upper limb paralysis after hemorrhagic stroke: a stroke-related epilepsy case. Acta Epileptol. (2025) 7:8. doi: 10.1186/s42494-024-00198-9, 40217410 PMC11960275

[23] Cai X Jiang J Zhou G Zhang Y. Mechanisms of Vagus nerve stimulation in improving motor dysfunction after stroke. Neuropsychiatr Dis Treat. (2024) 20:2593–601. doi: 10.2147/NDT.S492043, 39723115 PMC11669332

[24] Dawson J Liu CY Francisco GE Cramer SC Wolf SL Dixit A . Vagus nerve stimulation paired with rehabilitation for upper limb motor function after ischaemic stroke (VNS-REHAB): a randomised, blinded, pivotal, device trial. Lancet. (2021) 397:1545–53. doi: 10.1016/S0140-6736(21)00475-X, 33894832 PMC8862193

[25] Wei T Ge X Lu L Li J Xu P Wu Q. Efficacy and safety of vagus nerve stimulation on upper extremity motor function in patients with stroke: a meta-analysis of randomized controlled trials. NeuroRehabilitation. (2023) 53:253–67. doi: 10.3233/NRE-230106, 37694313

[26] Hasan MY Siran R Mahadi MK. The effects of Vagus nerve stimulation on animal models of stroke-induced injury: a systematic review. Biology (Basel). (2023) 12:12. doi: 10.3390/biology12040555, 37106754 PMC10136363

[27] Yang H Shi W Fan J Wang X Song Y Lian Y . Transcutaneous auricular vagus nerve stimulation (ta-VNS) for treatment of drug-resistant epilepsy: a randomized, double-blind clinical trial. Neurotherapeutics. (2023) 20:870–80. doi: 10.1007/s13311-023-01353-9, 36995682 PMC10275831

[28] Iovino P Dollaku H Alvaro R Pucciarelli G Rasero L Macchi C . Sleep quality patterns in patients with heart failure: a person-centred latent class analysis from a secondary analysis of the MOTIVATE-HF trial. BMJ Open. (2025) 15:e101950. doi: 10.1136/bmjopen-2025-101950, 40780724 PMC12336536

[29] Yan X Zhang X Huang C Jiang Y Wan C. Applications of transcranial direct current stimulation over vagus nerve on dysphagia after stroke. J Neurophysiol. (2025) 133:464–71. doi: 10.1152/jn.00588.2024, 39835801

[30] Riboldazzi G Spinazza G Beccarelli L Prato P Grecchi B D'Abrosca F . Effectiveness of expiratory flow acceleration in patients with Parkinson's disease and swallowing deficiency: a preliminary study. Clin Neurol Neurosurg. (2020) 199:106249. doi: 10.1016/j.clineuro.2020.106249, 33039853

[31] Kimberley TJ Cramer SC Wolf SL Liu C Gochyyev P Dawson J . Long-term outcomes of Vagus nerve stimulation paired with upper extremity rehabilitation after stroke. Stroke. (2025) 56:2255–65. doi: 10.1161/STROKEAHA.124.050479, 40329913

[32] Dawson J Engineer ND Cramer SC Wolf SL Ali R O'Dell MW . Vagus nerve stimulation paired with rehabilitation for upper limb motor impairment and function after chronic ischemic stroke: subgroup analysis of the randomized, blinded, pivotal, VNS-REHAB device trial. Neurorehabil Neural Repair. (2023) 37:367–73. doi: 10.1177/15459683221129274 36226541 PMC10097830

[33] Francisco GE Engineer ND Dawson J Kimberley TJ Cramer SC Prudente CN . Vagus nerve stimulation paired with upper-limb rehabilitation after stroke: 2- and 3-year follow-up from the pilot study. Arch Phys Med Rehabil. (2023) 104:1180–7. doi: 10.1016/j.apmr.2023.02.012, 37001842

[34] Wang Y He Y Jiang L Chen X Zou F Yin Y . Effect of transcutaneous auricular vagus nerve stimulation on post-stroke dysphagia. J Neurol. (2023) 270:995–1003. doi: 10.1007/s00415-022-11465-5, 36329182

[35] Abdullahi A Wong T Ng S. Effects and safety of vagus nerve stimulation on upper limb function in patients with stroke: a systematic review and meta-analysis. Sci Rep. (2023) 13:15415. doi: 10.1038/s41598-023-42077-2, 37723225 PMC10507009

[36] Li L Wang D Pan H Huang L Sun X He C . Non-invasive Vagus nerve stimulation in cerebral stroke: current status and future perspectives. Front Neurosci. (2022) 16:820665. doi: 10.3389/fnins.2022.820665, 35250458 PMC8888683

[37] Li JN Xie CC Li CQ Zhang GF Tang H Jin CN . Efficacy and safety of transcutaneous auricular vagus nerve stimulation combined with conventional rehabilitation training in acute stroke patients: a randomized controlled trial conducted for 1 year involving 60 patients. Neural Regen Res. (2022) 17:1809–13. doi: 10.4103/1673-5374.332155, 35017442 PMC8820701

